# An automated method for efficient, accurate and reproducible construction of RNA-seq libraries

**DOI:** 10.1186/s13104-015-1089-9

**Published:** 2015-04-03

**Authors:** Maria Tsompana, Sujith Valiyaparambil, Jonathan Bard, Brandon Marzullo, Norma Nowak, Michael Joseph Buck

**Affiliations:** Department of Biochemistry and Center of Excellence in Bioinformatics and Life Sciences, State University of New York at Buffalo, 701 Ellicott St., 14203 Buffalo, NY USA

**Keywords:** RNA-seq, Next generation sequencing, Chromatin, Epigenetics, Transcription

## Abstract

**Background:**

Integration of RNA-seq expression data with knowledge on chromatin accessibility, histone modifications, DNA methylation, and transcription factor binding has been instrumental for the unveiling of cell-specific local and long-range regulatory patterns, facilitating further investigation on the underlying rules of transcription regulation at an individual and allele-specific level. However, full genome transcriptome characterization has been partially limited by the complexity and increased time-requirements of available RNA-seq library construction protocols.

**Findings:**

Use of the SX-8G IP-Star® Compact System significantly reduces the hands-on time for RNA-seq library synthesis, adenylation, and adaptor ligation providing with high quality RNA-seq libraries tailored for Illumina high-throughput next-generation sequencing. Generated data exhibits high technical reproducibility compared to data from RNA-seq libraries synthesized manually for the same samples. Obtained results are consistent regardless the researcher, day of the experiment, and experimental run.

**Conclusions:**

Overall, the SX-8G IP-Star® Compact System proves an efficient, fast and reliable tool for the construction of next-generation RNA-seq libraries especially for trancriptome-based annotation of larger genomes.

## Findings

### Background

Deciphering the underlying determinants of transcriptional regulation in relation to cell differentiation, functional diversification, environmental signaling, and disease development remains a central question in biology today. Integration of expression data with knowledge on chromatin accessibility, histone modifications, DNA methylation, and transcription factor binding, has been instrumental for the unveiling of cell-specific local and long-range regulatory patterns, facilitating further investigation on the underlying rules of transcription regulation at an individual and allele-specific level. Current interest by large collaborative projects, such as the ENCODE [1], the NIH Roadmap Epigenomics Mapping Consortium [2,3], and the *C. elegans* and *D. melanogaster* modENCODE [4], has been placed on generating genome-wide gene expression maps to locate gene expression changes that accompany important developmental and disease development processes. The pairing of traditional expression assays with high-throughput sequencing (RNA-seq) has allowed the generation of genome-wide gene expression data with unparalleled specificity, throughput, and sensitivity delivering a detailed representation of the transcriptome.

However, full genome transcriptional gene characterization has been partially limited by the complexity and increased time-requirements of available RNA-seq library construction protocols. Here we report the successful application of the SX-8G IP-Star® Compact System (Diagenode) for the easy, rapid, and reproducible RNA-seq library construction of five *Mus musculus* (mouse) samples. Use of the SX-8G IP-Star® Compact System significantly reduced the hands-on time for RNA-seq library synthesis, adenylation, and adaptor ligation providing with high quality RNA-seq libraries tailored for Illumina high-throughput next-generation sequencing. Generated data exhibited high technical reproducibility compared to data from RNA-seq libraries synthesized manually for the same samples. Obtained results are consistent regardless the researcher, day of the experiment, and experimental run. Overall, the SX-8G IP-Star® Compact System proves an efficient and reliable tool for the construction of next-generation RNA-seq libraries especially for trancriptome-based annotation of larger genomes.

### Methods

**Figure 1:**
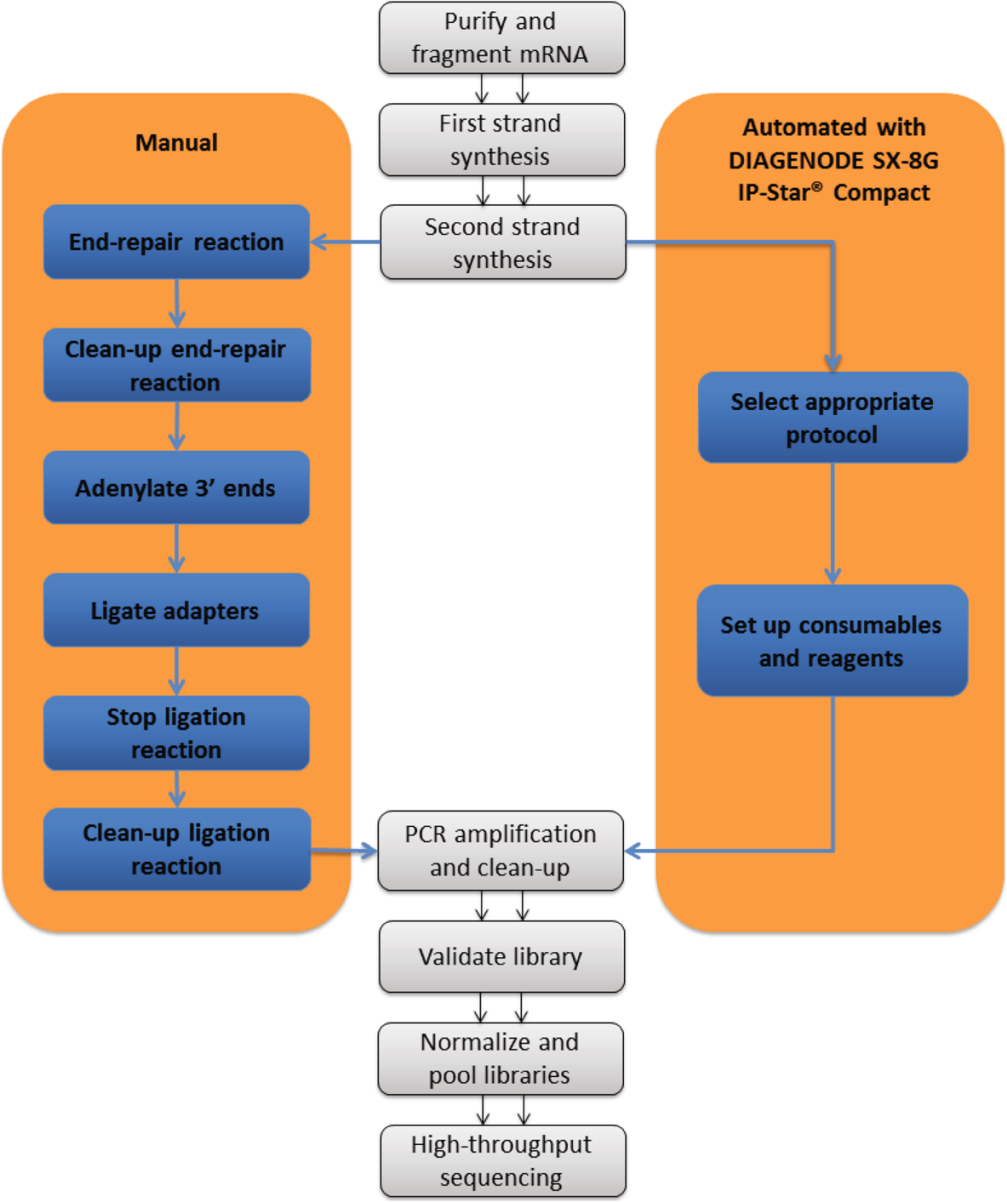
**A schematic representation of the sample preparation workflow.** The processes of the TruSeq™ RNA Sample Preparation v2 low sample (LS) protocol (Illumina) performed manually and adopted for automated use with the SX-8G IP-Star® Compact System are illustrated. The automated protocol minimizes the hands-on time required for the error-prone manual steps of RNA-seq library synthesis, adenylation, and adaptor ligation including all related clean up steps and allows experimental multitasking for the researcher in task.

A schematic step-wise representation of the two tested protocols is presented in Figure 1. Specifically, we tested application of the SX-8G IP-Star® Compact System for the construction of RNA-seq libraries of five mouse (Mm_1-5_Auto) samples in comparison to a manual protocol routinely used in our laboratory. The two protocols were compared using the same thermocycling machines and reagents. Total RNA integrity value following isolation was measured using the Agilent Technologies 2100 Bioanalyzer and was equal to eight for all tested samples. For the manual protocol mRNA preparation, library construction, and purification were done according to the TruSeq™ RNA Sample Preparation v2 low sample (LS) protocol (Illumina). Briefly, mRNA was extracted from 0.2 μg of total RNA for each sample using 5 min incubation with 50 μl of RNA Purification Beads (TruSeq™ RNA Sample Preparation Kit v2; Illumina) at 65°C, followed by 5 min incubation at room temperature. Following washing and elution of the mRNA denaturation reaction, mRNA was fragmented using 8 min incubation with 19.5 μl of the Elute, Prime, Fragment Mix (TruSeq™ RNA Sample Preparation Kit v2) at 94°C. First Strand Synthesis was performed using thermocycling with 8 μl of First Strand Master Mix (TruSeq™ RNA Sample Preparation Kit v2) and SuperScript II Reverse Transcriptase (Invitrogen) at 25°C for 10 min, 42°C for 50 min and 70°C for 15 min. For second strand synthesis samples were incubated with 25 μl of Second Strand Master Mix (TruSeq™ RNA Sample Preparation Kit v2) at 16°C for 1 hour. Reactions were cleaned up with Agencourt AMPure XP beads (Beckman Coulter Genomics). Libraries were end-repaired, adenylated at the 3’ end, ligated with adapters and amplified according to the TruSeq™ RNA Sample Preparation v2 LS protocol. Constructed RNA-seq libraries were purified with Agencourt AMPure XP beads and quantified using the Quant-iT™ PicoGreen® ds DNA Assay Kit (Invitrogen) and the KAPA Library Quantification Kit (KAPABIOSYSTEMS) using qPCR. Library quality control was performed with the Agilent Technologies 2100 Bioanalyzer. Libraries were normalized and pooled using the TruSeq™ Cluster Kit v3 (Illumina) based on the qPCR values. Pooled samples were sequenced using the HiSeq 2500 v3 sequencer (Illumina). For the automated protocol the assay was performed as above except that the most time-consuming stage of library preparation, synthesis, and adaptor ligation was performed using the SX-8G IP-Star® Compact System. The only required actions for this purpose were to select the appropriate Diagenode Library Preparation protocol (Illumina_TruSeq_DNA_SamplePrep_v2) for the corresponding sample number and to set up the necessary reagents and consumables following the robot’s user-friendly and simple interface.

RNA-seq data generated using the manual and automated protocols were aligned against the *Mus musculus* GRCm38/mm10 genome using TopHat 2.0.7 [5]. Following extraction of known transcripts, based on the most parsimonious trancriptome assembly, Fragments Per Kilobase of transcript per Million mapped reads (FPKM) values for each sample processed with the automated (Mm_1-5_Auto) and manual protocol (Mm_1-5_Man) were generated using the open-source software package Cufflinks 2.1.1 [6,7] to estimate relative transcript abundance. Transcripts from unexpressed genes with FPKM values equal to or less than 0.01 were excluded from subsequent analysis. Heat map plots and correlation coefficient values (r^2^, linear regression model) based on FPKM values of each sample and corresponding technical replicate were generated using the statistical language R. Data visualization, density distribution of FPKM values and cluster analysis were performed using the CummeRbund 2.7.1 R package (http://compbio.mit.edu/cummeRbund/).

### Results

Application of the SX-8G IP-Star® Compact System for the RNA-seq library construction of five mouse samples, significantly reduced the amount of hands-on time required for the most time-demanding stages of library synthesis, adenylation, and adaptor ligation including all related clean up steps. Specifically, manual library construction with the protocol routinely used in our laboratory typically takes an average of four hours of hands-on time whereas Diagenode automated library construction with the same reagents and samples required only 30 minutes. This corresponds to a 8-fold decrease in the amount of time the researcher has to be directly involved with the procedure, offering substantial flexibility for experimental multitasking.

**Figure 2:**
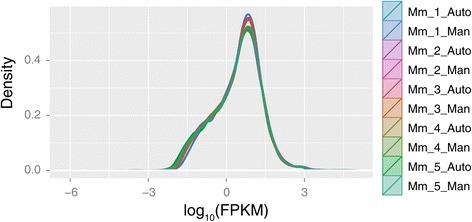
**Comparison of distributions of FPKM values.** Density distributions of FPKM values created using the CummeRbund 2.7.1 R package, support high data concordance among samples and corresponding technical replicates. Mm_1-5_Auto and Mm_1-5_Man correspond to mouse samples processed with the automated and manual protocols respectively.

**Figure 3:**
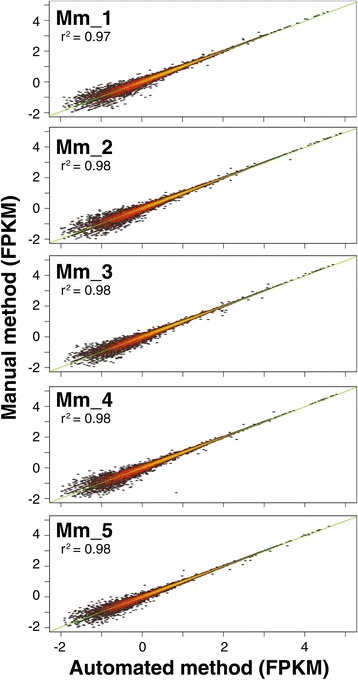
**Correlation analysis of FPKM values.** Heat map plots generated based on FPKM data from samples processed with the SX-8G IP-Star® Compact System (Mm_1-5_Auto) and their corresponding technical replicates (Mm_1-5_Man). Transcripts from unexpressed genes were excluded using a cut-off FPKM value equal to or less than 0.01. Correlation coefficient r^2^ between each sample and technical replicate was estimated using the linear regression model in R and ranged from 0.97-0.98, confirming the high technical reproducibility between the two tested protocols.

**Figure 4:**
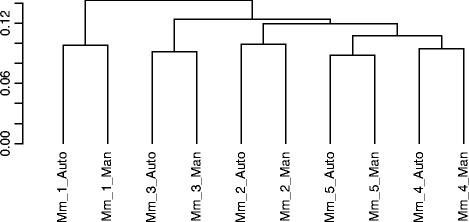
**Cluster analysis of FPKM values.** Analysis exhibits tight clustering of the tested samples (Mm_1-5_Auto) with the corresponding technical replicates (Mm_1-5_Man) confirming high technical reproducibility between the two protocols under study.

Notably, generated data with the automated protocol exhibited high technical reproducibility compared to data from RNA-seq libraries synthesized manually for the same samples regardless operator and experimental run. Specifically, density distributions of FPKM values demonstrated high data concordance among samples and technical replicates (Figure 2). Correlation coefficient values r^2^ obtained using the linear regression model in R for the five mouse samples and corresponding technical replicates ranged from 0.97-0.98, confirming that the SX-8G IP-Star® Compact System can be reliably used for the efficient and accurate construction of RNA-seq libraries (Figure 3). Cluster analysis illustrated tight clustering between samples and technical replicates, further supporting high technical reproducibility between the two tested protocols (Figure 4).

### Conclusions

Overall, the SX-8G IP-Star® Compact System proves an efficient, reliable and accurate tool for the construction of next-generation RNA-seq libraries, especially for trancriptome-based annotation of larger genomes. We foresee that incorporation of this technology in Next-Generation Sequencing Cores or Genomics Laboratories will prove an indispensable tool for high-throughput RNA-seq library construction, significantly saving on-hands experimentation time, related costs and error-prone manual steps. Added benefits of the automated protocol include ease of operation and generation of consistent data regardless of human variability and experimental run. Adaptation of this technology should support the unveiling of the mechanisms governing differential gene expression and transcription processing genome-wide, leading to a better understanding of genetic and epigenetic regulation and inheritance in a time-efficient manner.

## Abbreviations

RNA-Seq: Ribonucleic acid next-generation sequencing
mRNA: Messenger ribonucleic acid
qPCR: Quantitative polymerase chain reaction
FPKM: Fragments per kilobase of transcript per million mapped reads
